# Supplementary material for: Ecotoxicity of Heteroaggregates of Polystyrene Nanospheres in Chironomidae and Amphibian
Source: Nanomaterials (Basel). 2022 Aug 8;12(15):2730. doi: 10.3390/nano12152730 (PMC9370236; doi:10.3390/nano12152730)
Supplement: Supplementary file 1 [file nanomaterials-12-02730-s001.zip › nanomaterials-1794464-supplementary.pdf]

nanomaterials

Supplementary Materials

Ecotoxicity of Heteroaggregates of Polystyrene Nanospheres in Chironomidae and Amphibian

Florence Mouchet [1,*], Laura Rowenczyk [2], Antoine Minet [1], Fanny Clergeaud [1], Jérôme Silvestre [1], Eric Pinelli [1], Jessica Ferriol [1], Joséphine Leflaive [1], Loïc Ten-Hage [1], Julien Gigault [3], Alexandra Ter Halle [2] and Laury Gauthier [1]

1 Laboratoire d'Ecologie Fonctionnelle et Environnement, UMR 5245 CNRS, Université Paul Sabatier, 31062 Toulouse, France
2 Laboratoire des Interactions Moléculaires et Réactivité Chimique et Photochimique (IMRCP), UMR CNRS 5623, Université Paul Sabatier, Bâtiment 2R1, 3ème étage, 118, Route de Narbonne, 31062 Toulouse, France
3 Laboratoire Takuvik, CNRS, Laval University, Avenue de la Médecine, Quebec QC 1045, Canada
* Correspondence: florence.mouchet@ensat.fr

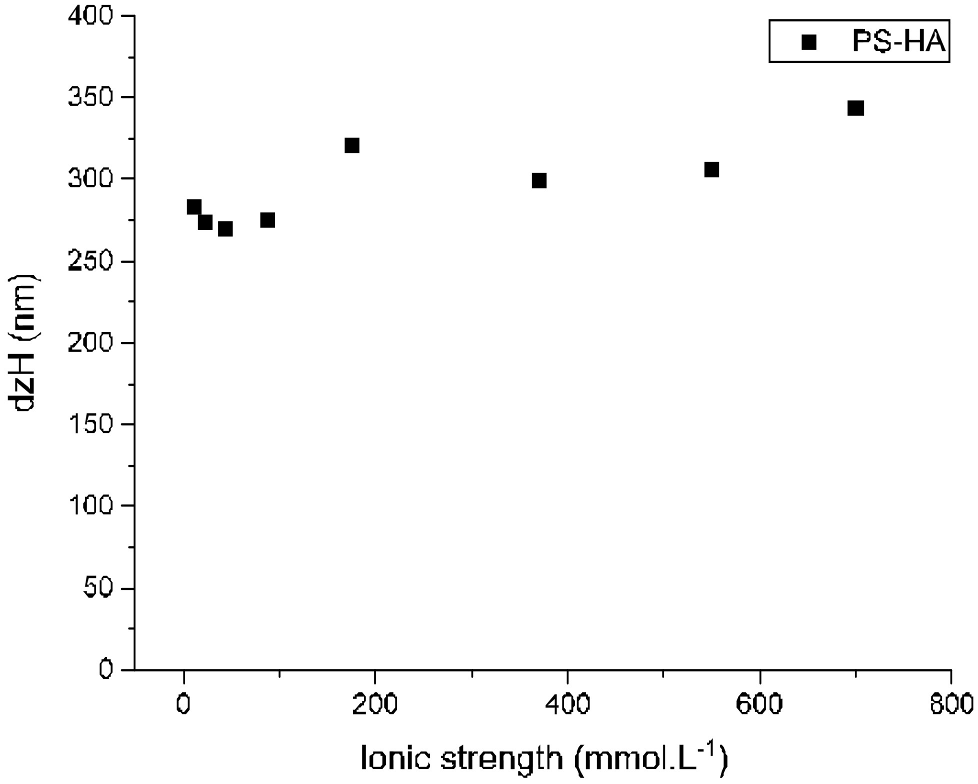

Figure S1. Stability of the PS-HA with ionic strength. The PS-HA were initially in water with NaCl (I = 700 mmol·L^{-1}). They were diluted by adding deionized water, and DLS measurements were performed in situ. The cumulants and SBL methods showed that the heteroaggregates were stable during dilution.

Table S1. Characterization of exposition media prepared with tap water (Ionic strength about 9 mM). Exposition media were prepared with numerous salts dissociated into ions when dissolved in water. Their pH was slightly basic, and their conductivity was above 1 mS/cm and similar. It is worth noting that the ionic strength of the tap water had to be considered in calculating the ionic strength. Thanks to data from "Eau de Paris" (http://www.eaudeparis.fr/leau-au-quotidien/une-eau-de-qualite/ accessed on 31 October 2018), the ionic strength of the tap water was found to be 9 mmol·L^{-1}. This value was added to the media ionic.

	T (°C)	pH	Conductivity (mS/cm)	Ionic strength (I) (mmol·L^{-1})
Chironomus	16.6	8.23	0.582	8.95 + 9.00 = 17.95
Amphibian	16.1	8.05	0.897	17.72 + 9.00 = 26.72
